# Bilateral segmentectomies using virtual-assisted lung mapping (VAL-MAP) for metastatic lung tumors

**DOI:** 10.1186/s40792-017-0379-y

**Published:** 2017-09-18

**Authors:** Keita Nakao, Masaaki Sato, Jun-ichi Nitadori, Jun Nakajima

**Affiliations:** Department of Thoracic Surgery, Graduate School of Medicine, University of Tokyo, 7-3-1 Hongo, Bunkyo-ku, Tokyo, 113-8655 Japan

**Keywords:** Thoracoscopic surgery, Metastatic pulmonary tumor, Virtual-assisted lung mapping (VAL-MAP)

## Abstract

**Background:**

Virtual-assisted lung mapping (VAL-MAP) has been used not only in wedge resection but also in segmentectomy for hardly palpable lung nodules. We herein report a case of bilateral segmentectomy using VAL-MAP with chronological change of pulmonary function test results.

**Case presentation:**

A 50-year-old female was found to have a colorectal cancer with pulmonary nodules in both sides of the lungs considered as synchronous lung metastases. After sigmoidectomy for primary cancer and chemotherapy, treatments for small nodules in both sides of the lungs were planned. Most nodules were small and supposed to be impalpable. We performed thoracoscopic segmentectomy of right S8 with the aid of VAL-MAP and, after 2 months, combined subsegmentectomy of left S8a and 9a and wide wedge resection of left S8b with the aid of VAL-MAP. All nodules suspected of lung metastases were successfully resected with adequate margins, and the decrease in pulmonary function was minimal compared with predicted postoperative forced vital capacity (FVC) and forced expiratory volume (FEV) 1.0 calculated by the numbers of subsegments.

**Conclusions:**

Bilateral segmentectomies of small impalpable metastatic tumors were performed successfully with the aid of VAL-MAP.

## Background

At first, virtual-assisted lung mapping (VAL-MAP) was developed to help to identify a small hardly palpable ground grass nodule represented by less invasive adenocarcinoma [1, 2]. As the utility in confirming intersegmental planes in segmentectomy has also been recognized [3, 4], segmentectomy using VAL-MAP has been conducted as a part of a clinical trial in multiple institutions in Japan (UMIN 000008031) and the outcome has recently been published [5]. This report shows a case of bilateral segmentectomies using VAL-MAP with chronological change of pulmonary function test results.

## Case presentation

**Fig. 1:**
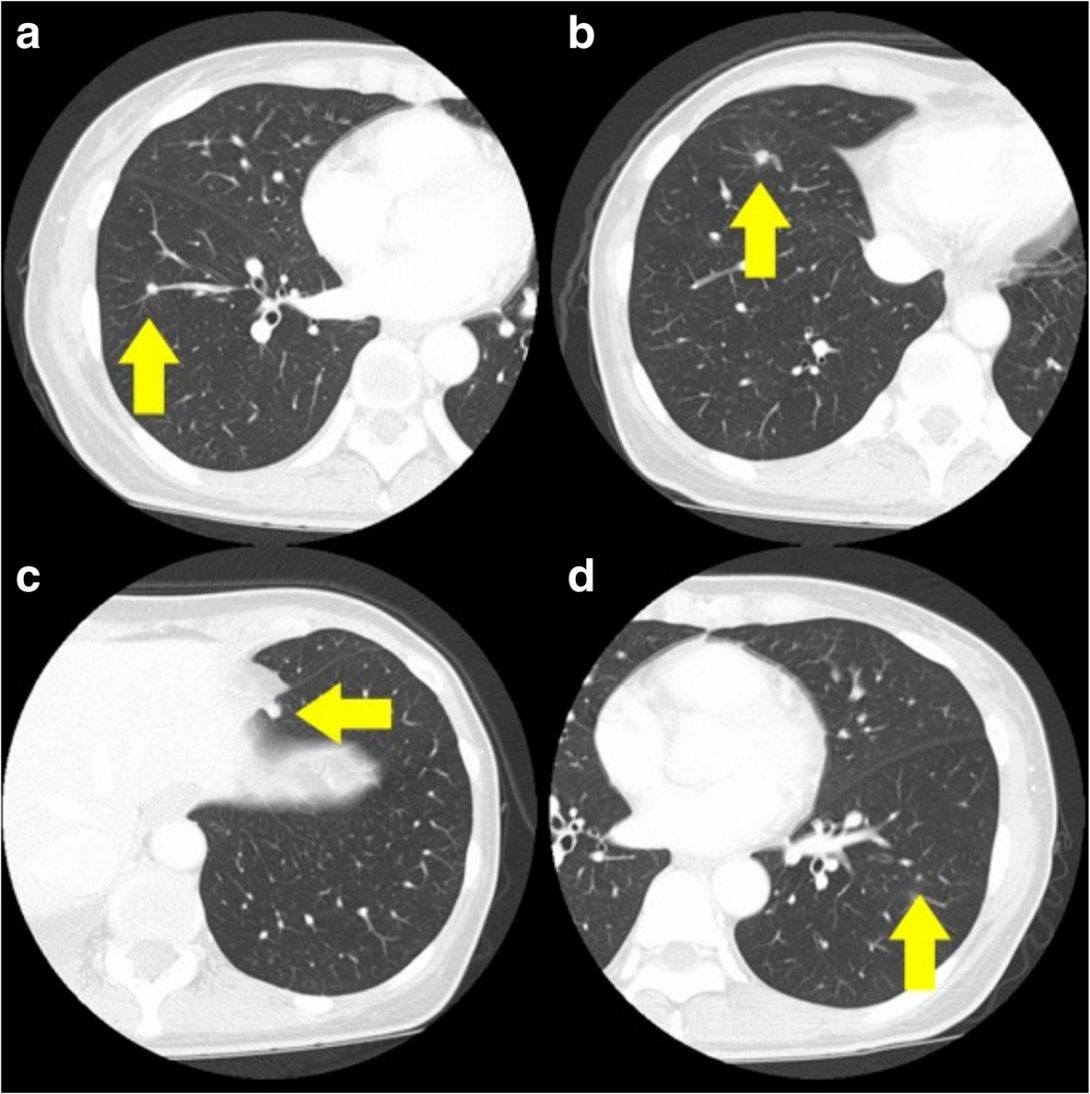
Preoperative CT images of abnormal nodules. **a** Right S8a (5 mm). **b** Right S8b (8 mm). **c** Left S9 (5 mm). **d** Left S8 (3 mm)

A 50-year-old female with no particular past medical history was diagnosed as sigmoid colon cancer with synchronous pulmonary metastases in both sides of lungs. She underwent laparoscopy-assisted sigmoidectomy and four courses of adjuvant chemotherapy with capecitabine 2400 mg at day 1 and oxaliplatin 170 mg from day 1 to day 14. The response of adjuvant chemotherapy was stable disease and neither shrinking nor increasing of pulmonary lesions was observed. She was referred to our department for surgical treatment for small pulmonary metastasis in both sides of lungs. There were four nodules located in the right segment 8a (S8a), right S8b, left S8, and left S9 (Fig. 1a–d).

We planned to perform two-staged operations: primarily the segmentectomy of right S8 with the aid of VAL-MAP followed by the secondary combined segmentectomy of left S8a + 9a and wide wedge resection of left S8b using VAL-MAP.

**Fig. 2:**
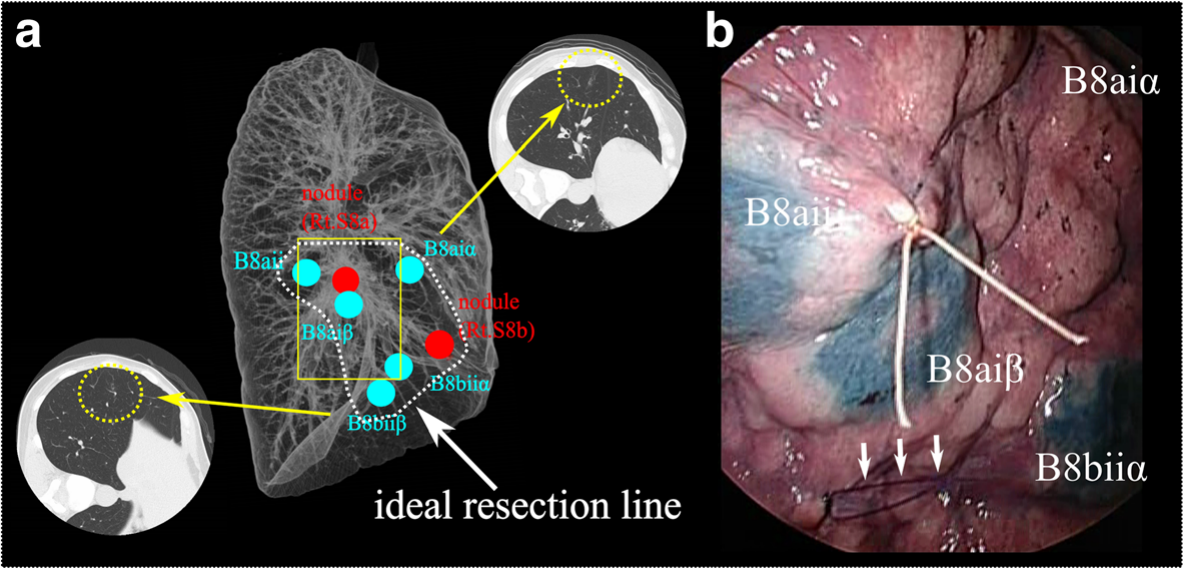
**a** Three-dimensional configuration of the CT for “mapping” for the first operation. **b** A thoracoscopic view corresponding to the yellow square in **a**. The “standing stitch” with blue suture (white arrows) near a marking spot indicates the resection line between S8 and S9 and a silk stitch just above the nodule in right S8a. The geometric information of ideal resection line was obtained from marking spots and blue sutures were placed along the resection lines

At the time of initial admission to our department, we selected five points for markings between right S8 and S9 and one point on the diaphragmatic surface using Synapse Vincent® (Fujifilm Medical; Tokyo, Japan) as described previously in details [6]. Bronchoscopic dye injection to peripheral branch of right B8aii, B8aiα, B8aiβ, B8biiα, and B8biiβ was performed in the morning on the day of surgery followed by another chest computed tomography (CT) and three-dimensional reconstruction, which were conducted to confirm the location of “mapping” in routine [7]. In the 3D configuration, we confirmed how the “mapping” looked and adjusted the surgical plan (Fig. 2a).The details of thoracoscopic VAL-MAP-assisted stapler-based segmentectomy were reported previously [3].

The operation was started with general anesthesia and isolated lung ventilation in left lateral decubitus position. We made three ports in 4th intercostal space of middle axillary line (12 mm), 5th intercostal space of posterior axillary line and 7th intercostal space of anterior axillary line. When observed with a thoracoscope, every marking was clearly visible and then we made five stiches along the planned resection lines. Four stiches were placed close to marking spots and one at the border with S7 at the diaphragmatic surface along the scheduled segmentation lines with 4–0 PROLENE® (Johnson & Johnson; New Jersey, USA) and also made another marking above the nodule with silk (Fig. 2b). Although each dye marking was located on the lung surface at the periphery of the branchial branch (i.e., inside of the segment), the appropriate intersegmental lines and the locations of blue sutures were determined based on the geometric information obtained from the “lung map.” The lung parenchyma between S6 and S8 was cut with staplers along the intersegmental lines. A8a, A8b, B8, and V8a were cut sequentially. The lung parenchyma between S8 and S7 was separated with staplers, and the resected S8 was taken out from thoracic cavity. Polyglycolic acid mesh and fibrin glue were used to seal minor air leakage from the resected lung parenchyma. Two chest tubes were inserted into the thoracic cavity and the operation was finished. The operation took about 4 h and the blood loss was 30 ml. The postoperative course was uneventful, and the chest tubes were removed 2 days after the surgery. She was discharged 6 days after the surgery.

Histologically, intestinal-type differentiated adenocarcinoma were observed and these were histological images similar to that of a prior sigmoid colon cancer, which were considered to be lung metastases of the same cancer. The smallest excision margin was 20 mm and negative.

**Fig. 3:**
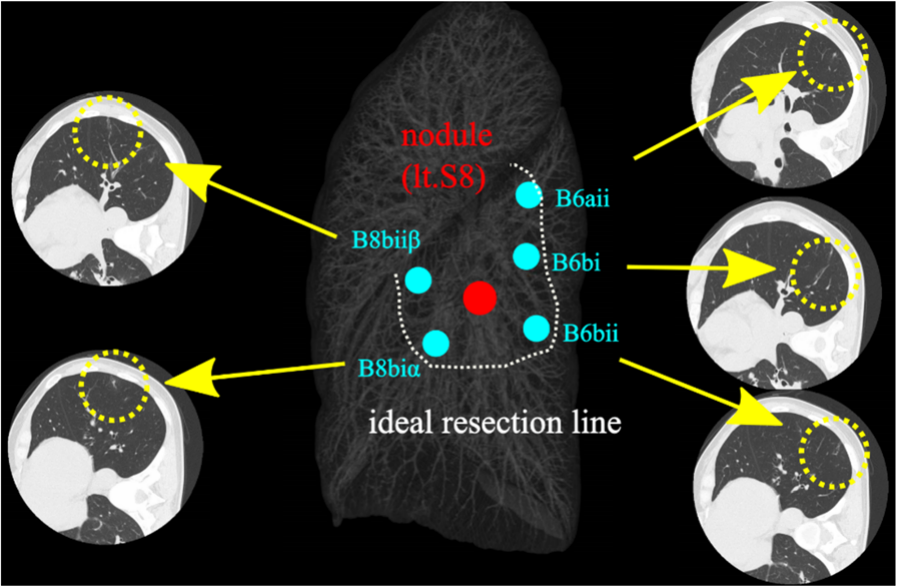
Three-dimensional configuration of the CT for “mapping” for the second operation

We planned to conduct the other side operation on schedule 2 months after the first surgery without degradation of physical function even after the initial surgery. Her pulmonary function 1 month after first surgery was acceptable for the second surgery (Table 1). Another chest CT scan revealed no new nodules. We decided to conduct thoracoscopic left S8a and S9a combined subsegmentectomy and wide wedge resection of left S8b. To ensure resection margins, once again, VAL-MAP was scheduled before planned operation. We marked five points in the peripheral branch of left B6aii, B6bi, B6bii, B8biiβ, and B8biα with the intention of identifying the nodule and ideal intersegmental lines. The chest CT was examined just after bronchoscopy and 3D configuration of the CT for “mapping” was made (Fig. 3). After the five blue sutures near the marking spots and one silk stitch were placed and the main vessels were resected, the bronchi were dissected and the S8a and S9a combined subsegmentectomy was done in a similar manner as described above. The other nodule in S9b was identified easily from the lung surface, and wide wedge resection was completed with staplers. One chest tube was inserted, and the operation took about 3 and a half hours with minimal blood loss. The postoperative course was uneventful, and the chest tube was removed 2 days after surgery. She was discharged 5 days after the surgery. The pathological finding of the lesion on the left S9 was consistent with the findings in the lung metastasis of colon cancer, and the lesion on the left S8 was the lymph node in the lung. The excision margin from the lesion on the left S9 was 25 mm and negative.

**Table 1:** Results of respiratory function tests over time and predicted postoperative function calculated by the number of resected subsegments

	Pre-operation	1 month after 1st operation	1 year after 2nd operation
FVC (L)	3.22	2.42	2.84
ppoFVC (L)		3.07	2.91—wedge resection
%FVC (%)	123.7	93.5	99.7
FEV1.0 (L)	2.28	1.89	1.98
ppoFEV1.0 (L)		2.17	2.06—wedge resection
FEV1.0% (%)	70.67	77.06	69.92

No recurrent disease was identified about a year and a half after the second surgery. She returned to work without any symptom related to bilateral segmentectomies. Changes in respiratory function tests over time and corresponding predicted postoperative FVC and FEV1.0 calculated by the numbers of subsegments [8] are shown in the Table 1.

## Conclusions

We report a case of bilateral segmentectomy for pulmonary metastases with the aid of VAL-MAP. Several studies had revealed patients with metastases limited to the lungs may benefit from pulmonary metastasectomy [9]. Wedge resection is often performed for a peripheral nodule, but more extensive resection is required when wedge resection would not achieve complete resection [10]. To preserve as much functional lung tissue as possible, segmentectomy should be the first option if all lesions cannot be removed via wedge resection [10]. In the present case, three out of four lesions were deeply located so that wedge resection was considered to be difficult.

In segmentectomy, identification of appropriate resection lines is critical. There are several methods to identify intersegmental planes intraoperatively, such as VAL-MAP, usage of inflation-deflation lines, and injection of indocyanine green (ICG) into selected pulmonary artery or bronchus [11–13]. To use inflation-deflation line, the condition of expansion and collapse is not necessarily clear, the surgical field is often interfered with the procedure. Intravenous or transbronchial injection of ICG can be used only if expensive infrared light thoracoscopic device is available. Compared with these two methods, VAL-MAP has advantages in the easy application to minimally invasive thoracoscopic surgery without special instrument. The “standing stitches” placed at the beginning are clearly visible throughout surgery even under limited vision [3]. In limited resection for malignant tumor, it is important to obtain sufficient resection margins; another advantage of VAL-MAP is its applicability to extended segmentectomy beyond anatomical segments [3].

The result of pulmonary function tests in the present case demonstrated reasonable preservation of pulmonary function even after bilateral segmentectomies using staplers with the aid of VAL-MAP. After the first operation, the measured FVC and FEV1.0 were worse than those calculated predicted postoperative values probably due to the short time period after surgery [8]. Although the second operation contained a wide wedge resection, the actual postoperative pulmonary functional parameters measured 1 year after the second operation were almost equal to the predicted ones, given that the volume of wide-wedge resection was similar to that of a single subsegment.

In conclusion, we conducted bilateral stapler-based segmentectomies with the aid of VAL-MAP without any postoperative complications and the decrease of pulmonary function was within the range of prediction.

## Abbreviations

CT: Computed tomography
FEV: Forced expiratory volume
FVC: Forced vital capacity
ICG: Indocyanine green
ppoFEV: Predicted postoperative forced expiratory volume
ppoFVC: Predicted postoperative forced vital capacity
VAL-MAP: Virtual-assisted lung mapping
